# Satisfaction Levels of Young Urban Residents With Public Sports Services in China During the COVID-19 Epidemic

**DOI:** 10.3389/fpsyg.2022.905417

**Published:** 2022-06-10

**Authors:** Min Liu, Dawei Yu, Baoxia Chen, Zhusheng Wu, Zichao Chen, Yuanfang Pan, Shiying Gao, Zexia Jia, Shanshan Li, Yong Jiang

**Affiliations:** ^1^School of Sports Science, Qufu Normal University, Qufu, China; ^2^School of Physical Education, Huzhou University, Huzhou, China; ^3^School of Physical Education, Sichuan University, Chengdu, China; ^4^School of Economics, Sichuan University, Chengdu, China; ^5^School of Physical Education, Shenzhen University, Shenzhen, China

**Keywords:** public sports service, urban residents, satisfaction, COVID-19, structural equation model

## Abstract

The extensive changes in public and private life caused by the ongoing COVID-19 epidemic in China have created a “new normal.” To understand urban residents’ satisfaction with public sports services under these conditions and to identify factors that influence satisfaction, urban residents in several major Chinese cities were randomly sampled with an online questionnaire to measure their level of satisfaction with public sports services in China during the COVID-19 epidemic. With the response rate of 87.9%, 703 valid questionnaires out of 800 questionnaires distributed were analyzed. A structural equation model was constructed where health literacy and the residents’ expectations were independent variables, residents’ participation was a mediating variable, and residents’ satisfaction was the dependent variable. Cronbach’s α test and Kaiser-Meyer-Olkin test confirmed that the questionnaire was reliable and valid. Survey results suggest that young urban residents’ participation in public sports services is influenced by personal health literacy and residents’ expectations. Participation serves a mediating role between health literacy and residents’ satisfaction, but not between residents’ expectations and their satisfaction. Young urban residents’ satisfaction with public sports services may be improved by increasing access to health information, improving hardware and software platforms to support those services, and catering the services to the interests of the population.

## Introduction

The COVID-19 corona virus epidemic has greatly impacted the lives of many people. As of May 7, 2020, the restrictions put in place in high-risk areas in China to limit the spread of disease have been removed, and most aspects of life have gradually returned to normal. To a certain extent, the epidemic has enhanced the public’s awareness of physical exercise, and the demand for sports service has correspondingly increased (Kaur et al., 2020; Rao and Joshi, 2020), which may lead to the increase of the need for urban public sports services. As part of the initiative “Healthy China 2030” (The Central Committee of the Communist Party of China and the State Council of China, 2016), the government aims to strengthen the public sports service system to meet the surge in urban residents’ need for physical exercise and fitness, thereby improving their health and happiness. Specific measures include strengthening the construction of cycling trails, national fitness centers, sports parks, community multi-functional stadiums and other venues and facilities (Healthy China 2030, 2016). Therefore, this manuscript focused on the public sports service opened for free of charge or at low charges, which are flexible, adaptable, people-centered and could cover a wider population.

User satisfaction is the fundamental criterion for evaluating the performance of public sports services. Therefore, this manuscript investigated the satisfaction of urban residents with public sports services under a “new normal” in which preventive measures to keep the COVID-19 epidemic under control continue to limit public and private life. We constructed a model to analyze the influence of various factors on the satisfaction of urban residents with public sports services, which may help identify areas where services can be improved in cities and towns throughout China.

## Theoretical Background

Studies originating outside of China have established relatively mature theories and models about customer satisfaction. In contrast, for various reasons, research on public satisfaction in China is still in its early stages and has yet to formulate mature theory. Current models and expectations are still supported by foreign customer satisfaction theories. This may not provide the most reliable basis for research in China, especially when measuring satisfaction levels with public sports services, the provision of which differs substantially between China and western countries. Sports in western countries have been highly industrialized: beyond free public sport services, pay-for-use has become common because of financial problems and very cost-intensive maintenance of sport facilities (Ibsen and Levinsen, 2019; Zhou et al., 2020). On the contrary, China’s public sports services are provided by the government and free of charge (Yuan, 2016). This fundamental difference shows that Chinese scholars may use western theories and experiences as a guide – but not a template – to study resident satisfaction with public sports services.

In addition, most studies on satisfaction index have been conducted from the perspective of the service provider (Howat and Assaker, 2016), using objective measures such as sports facility service or sports information service (He and Xu, 2007; Martínez-Moreno and Suárez, 2016; Guo et al., 2018). Few studies have been conducted from the perspective of the users (Zhou et al., 2020), in this case young urban residents. Therefore, the present study collected data directly from residents to interrogate the influence of health literacy and expectations on their satisfaction and to determine mediating effects on their use of sport services. This innovative approach may generate results that can more readily be translated into actionable and quantifiable measures to improve satisfaction with public sports services.

## Concept of Satisfaction

Research on satisfaction began as early as the 1930’s in countries outside of China and in the field of psychology by Hoppe (1930) and Lewin (1936). Howard and Sheth (1969) defined customer satisfaction as a kind of psychological state reflecting the balance between the customer’s efforts and perceived gains. The KANO model, proposed by Kano et al. (1984), states that customer satisfaction depends on product quality. Parasuraman et al. (1988) proposed the Customer Satisfaction Index model, in which took users actual perceptions as the research object. This model has been developed extensively in the West, such as in Sweden, United States, and Europe (Cassel, 2000). Foreign studies on satisfaction are well grounded and mainly based on sports club. For example, Howat et al. distributed a questionnaire, including topics like behavioral intention, service quality and public satisfaction, to 5,283 respondents in Australia. The key factors affecting the customer’s perceived level of quality included facilities, core services, secondary services and staff. Importantly the customer’s perceived quality had a strong impact on their satisfaction and loyalty (Howat and Assaker, 2013; Avourdiadou and Theodorakis, 2014; Yanni et al., 2018; Lvarez-García et al., 2019). Tsuji et al. (2007) pointed out that the overall quality of sports services should be measured by evaluating the service quality of specific industries. Andrew et al. (2009) surveyed 218 spectators of small ice hockey leagues and found that sports facilities and service quality significantly impacted customer satisfaction.

Compared with foreign studies, Chinese studies on satisfaction started later, and most have investigated a specific industry service or product, rather than public sports services in general. Xiao (2012) indicated that the disabled generalizes demand for public sports services include sports funds, special sports facilities, special sports guidance services, sports regulations, and sports information. There is an urban-rural difference of satisfaction with public sports services, which primarily concentrates on sports consulting, but least on the availability and price (Guo et al., 2018). This manuscript has citizens in Chengdu urban areas as its research object and built an effective model of the government performance evaluation in public sports service (Zhao and Jiang, 2015). Public sport services in the areas of sport facility, sport organizations and sport activities had strong influences on participant satisfaction (Zhou et al., 2020). Jiang and Chen (2016) suggested that changing the sports concepts, promoting the construction of the sports facilities, leading the volunteer service activities and constructing the information communication network platform of the sports service can improve the supplies of the public sports services in the local cities.

## Model Construction and Hypotheses

**FIGURE 1:**
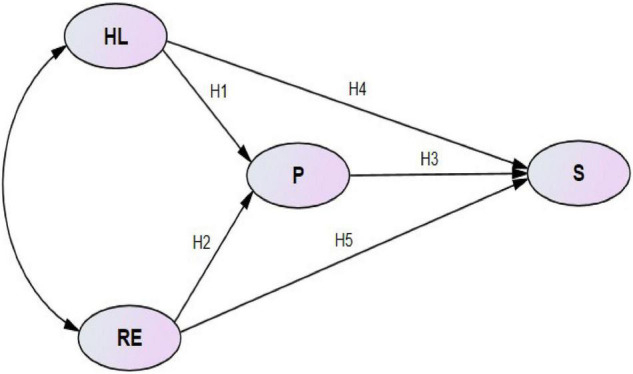
Conceptual model of factors affecting satisfaction of urban residents with public sports services. In this model, health literacy and residents’ expectations served as independent variables; residents’ participation, as an intermediary variable; and residents’ satisfaction, as the dependent variable. The hypotheses H1-H5 describing each relationship are indicated. HL, health literacy; RE, residents’ expectation; P, participation; S, satisfaction.

The present study proposes a model based on the European Customer Satisfaction Index Model (ECSI) and customer satisfaction theory. Customer satisfaction theory regards all kinds of public services or products as “commodities” and the entire public as “customers.” The quality of public services or products is measured in terms of the level of satisfaction reported by customers. The ECSI model involves the following dimensions: corporate image, customer expectation, perceived quality (hardware and software), perceived value, customer satisfaction, and customer loyalty. Corporate image, customer expectation, perceived quality, and perceived value determine customer satisfaction, thus affecting customer loyalty. Perceived quality is divided into hardware, referring to the quality composition of product or service attributes, and software, referring to the interactive experience between customers and service organizations in the service process. Our manuscript regards urban public sports services as “commodities” and urban residents with sports needs as “customers.” Based on the ECSI model, our analysis considers four dimensions: expectations, participation, satisfaction and health literacy. Residents’ awareness of health and demand of exercises will be enhanced being influenced by the COVID-19, so we believed that health literacy and residents’ expectation will have a great influence on participation and satisfaction. Therefore, a model is proposed that incorporates expectations and health literacy as independent variables, participation as an intermediary variable and satisfaction as a dependent variable (Figure 1). Health literacy refers to an individual’s comprehension of health issues, and it includes health knowledge and awareness (Seidel, 2020). We considered health literacy as an independent variable because we tried to find out how health literacy influence participation and satisfaction during the COVID-19 Epidemic.

In addition, research does not support an obvious correlation between perceived value and satisfaction, but does suggest public participation has a moderating effect on satisfaction and expectation as well as perceived quality and value. Therefore, our model includes resident participation instead of perceived value. These considerations led us to formulate the following hypotheses:

H1: Health literacy is positively correlated with residents’ participation.

H2: Residents’ expectations are positively correlated with their participation.

H3: Residents’ participation is positively correlated with their satisfaction.

H4: Health literacy is positively correlated with residents’ satisfaction.

H5: There is a positive correlation between residents’ expectations and satisfaction.

## Participants and Methods

### Participants

An online questionnaire was used to sample the urban residents in the provinces and municipalities directly under the Central Government in the inland areas of China. Of the 800 questionnaires randomly distributed, all were returned and 703 were valid. The relevant descriptive statistical results are shown in Table 1. The proportion of men and women in the survey sample were relatively balanced: 316 (45.4%) men and 387 (54.6%) women. Most respondents (84.3%) were 25–35 years old, and approximately 87% of those surveyed held undergraduate or graduate degrees. A majority (82.1%) of participants were from areas considered at low risk of COVID-19. Just over half (56.9%) of respondents reported that they used public sport services occasionally, 22.5% engaged frequently, and few never did. These results suggest that the survey sampling could provide reliable insights into young resident satisfaction with public sports services during the “new normal” of the COVID-19 epidemic.

**TABLE 1:** Demographic characteristics (N = 703).

Measure	Item	Number	%
Gender	Male	316	45
	Female	387	55
Age	≤25	451	64.2
	26–35	141	20.1
	36–45	63	9
	46–55	39	5.5
	≥56	9	1.3
Degree	Below junior college	46	6.5
	Junior college	45	6.4
	Undergraduate	396	56.3
	Graduate	216	30.7
Epidemic risk level	Highest	12	1.7
	High	29	4.1
	Moderate	85	12.1
	Low	160	22.8
	Lowest	417	59.3
Frequency of use of public sport services during the epidemic	Never	145	20.6
	Sometimes	400	56.9
	Often	158	22.5

### Methods

#### Questionnaire Design

In this study, a questionnaire was prepared based on the considerations in “Model Construction and Hypotheses” section, and a scale was formed based on previous studies. The questionnaire comprised 27 questions divided into four parts and a model question was shown for each subscale: health literacy (i.e., you have developed a healthy lifestyle and behavior habits), expectation (i.e., during the epidemic, you expect the government to pay more attention to public sports services), participation (i.e., after the epidemic, you participate in physical exercise more frequently), and satisfaction (i.e., you are satisfied with the sports facilities in your community). A 7-point Likert scale was used to respond to each question: 1 stood for strongly disagree; 2, disagree; 3, somewhat disagree; 4 neither disagree nor agree; 5, somewhat agree; 6, agree; and 7, strongly agree. Therefore, this scale can be used as a tool to measure the level of urban resident satisfaction with public sports service in the “new normal” of daily epidemic prevention.

#### Data Analysis

SPSS 22.0 (International Business Machines Corporation, New York, NY, United States) was used to analyze the reliability and validity of the data and exploratory factor analysis. AMOS 20.0 (International Business Machines Corporation, New York, NY, United States) was used to construct the structural equation model, which was modified to achieve better fitting and adaptability. The data were then analyzed to explore the relationships of health literacy, expectation and participation with urban residents’ satisfaction with public sports services.

## Results and Discussion

### Reliability Test

Cronbach’s α coefficient was used to test the reliability of the questionnaire. The Cronbach’s α for each scale (HL = 0.809, RE = 0.771, P = 0.735, S = 0.870) was greater than 0.7. The score for the questionnaire as a whole was 0.909, while the reliability of topic composition was between 0.387 and 0.882 (Table 2). These scores indicated that the questionnaire was credible as a tool for measuring satisfaction of urban residents with public sports services.

**TABLE 2:** Measurement model.

		Unstd.	S.E.	Z-Value	Std.	SMC	CR	AVE
HL	H1	1			0.819	0.671	0.810	0.588
	H2	0.814	0.048	**16.884**	0.722	0.521		
	H3	0.899	0.052	**17.199**	0.757	0.573		
RE	R1	1			0.629	0.396	0.777	0.541
	R2	1.468	0.108	**13.621**	0.850	0.723		
	R3	1.367	0.096	**14.227**	0.710	0.504		
P	P1	1			0.734	0.539	0.737	0.485
	P2	0.960	0.077	**12.471**	0.727	0.529		
	P3	0.879	0.071	**12.337**	0.622	0.387		
S	S1	1			0.939	0.882	0.877	0.707
	S2	0.937	0.034	**27.197**	0.869	0.755		
	S3	0.736	0.035	**20.989**	0.695	0.483		

*Values with a statistical significance of P < 0.001 are boldfaced.Unstd., unstandardized estimate; S.E., standard error; Std., standardized estimate; SMC, squared multiple correlations; CR, component reliability; AVE, average variance extracted value; HL, health literacy; RE, residents’ expectation; P, participation; S, satisfaction.*

### Validity Test

The Kaiser-Meyer-Olkin (KMO) test and Bartlett’s test of sphericity were used to assess that the topic composition of the questionnaire accurately tested our hypotheses. The KMO was 0.919 (P < 0.001), which is above the cut-off proposed by Kaiser, indicating that the data were valid (it is suitable for factor analysis when the KMO greater than 0.9) (Kaiser and Rice, 1974). Bartlett’s test showed that sig <0.001, which meant the date is suitable for factor analysis.

#### Discriminant Validity

For satisfaction and resident expectations, the average variance extracted (AVE) was higher than the correlation with other factors (Table 3). Conversely, Pearson correlation coefficients for resident participation and health literacy were slightly larger than the corresponding AVEs. This shows less good convergence validity for resident participation, yet all variables showed roughly comparable validity. Therefore, the overall model showed good discriminant validity.

**TABLE 3:** Discriminant validity.

	AVE	S	P	RE	HL
S	0.707	**0.841**			
P	0.485	0.416	**0.696**		
RE	0.541	0.068	0.608	**0.736**	
HL	0.588	0.274	0.830	0.614	**0.767**

*Boldface numbers are square-root AVEs; other numbers are Pearson correlation coefficients of the plane.AVE, average variance extracted; S, satisfaction; P, participation; RE, residents’ expectation; HL, health literacy.*

#### Convergent Validity

As shown in Table 2, the measurement model and the questionnaire topics were significant in parameter estimation (P < 0.001). The reliability of the topics was greater than 0.6 under standardization, and the SMC (squared multiple correlations) values were greater than 0.36, indicating that the topic of sports service satisfaction itself has good reliability. The CR (component reliability) values were above 0.7, showing good internal consistency among all four factors. All AVEs (average variance extracted value) were greater than 0.5, showing good convergent validity; residents’ participation was quite close to 0.5 (0.485), which is acceptable. Therefore the model showed good convergent validity and reliability, and the dimensions and topics were strongly related to one another.

### Model Evaluation

#### Model Fitness

The fit index of the model can verify its reliability and accuracy. The chi-squared test revealed that the value is not small enough to be considered good but can be accepted (Table 4). Since the chi-squared value of the model is affected by the number of samples and estimated parameters, the value was divided by the degrees of freedom. This analysis indicated that the model fit the data well. The same was indicated by the root mean square error of approximation and other fit indexes.

**TABLE 4:** Analysis of SEM model fit to the data.

Index of fit	Value	Quality criterion	References
χ^2^	249.35	Smaller is better	Maiti and Mukherjee, 1990; Schermelleh-Engel et al., 2003
χ^2^/df	4.987	3–5	Maiti and Mukherjee, 1990; Hooper et al., 2008
GFI	0.946	>0.90	Jöreskogn and Sörbom, 1981; Schermelleh-Engel et al., 2003
AGFI	0.916	>0.90	Jöreskogn and Sörbom, 1981; Schermelleh-Engel et al., 2003
RMSEA	0.075	<0.08	Steiger and Lind, 1980; Hooper et al., 2008
CFI	0.947	>0.90	Bentler, 1990; Hu and Bentler, 1999

*SEM, structural equation model; χ^2^, cmin; χ/df, cmin/degree of freedom; GFI, goodness-of-fit index; AGFI, adjusted goodness-of-fit index; RMSEA, root mean square error of approximation; CFI, comparative fit index.*

#### Hypothesis Testing

The model tested five hypotheses (Figure 1), which were assessed by analyzing the regression coefficients of the corresponding paths in the model (Table 5).

**TABLE 5:** Hypothesis testing based on the structural equation modeling (N = 703).

Hypothesis	Path	Path coefficient	Critical ratio	Supported?
H1	HL → P	0.718	11.814^a^	Yes
H2	RE → P	0.176	2.920^b^	Yes
H3	P → S	0.878	5.403^a^	Yes
H4	HL → S	−0.167	−1.157	No
H5 :	RE → S	−0.384	−4.173^a^	No

*^a^P < 0.00; ^b^P < 0.05.HL, health literacy; RE, residents’ expectation; P, participation; S, satisfaction.*

If H1 and H2 were true, then health literacy and residents’ expectation should each positively influence residents’ participation. If H3 were true, then residents’ participation should positively impact residents’ satisfaction. The path coefficient of health literacy for residents’ participation was 0.718, the critical ratio was 1.814, and the P value was significant, supporting that health literacy had an impact on residents’ participation. Thus, higher health literacy was associated with higher frequency and longer duration of sports participation. Residents’ expectations also had a significant impact on residents’ participation: the greater residents’ expectations were, the more willing they were to participate in public sports services. Residents’ participation also significantly affected residents’ satisfaction with public sports services, ultimately promoting further participation.

H4 and H5 were not supported by the data. In other words, health literacy and residents’ expectations did not affect residents’ satisfaction directly, but rather indirectly through their participation.

#### Verification of Intermediary Effects

The bootstrap confidence interval method was used to test the significance of mediating effects. It was possible that residents’ participation mediated the relationship of residents’ satisfaction with health literacy and residents’ expectations. Indeed, our analysis showed that health literacy indirectly affected residents’ satisfaction via their participation (Table 6). In contrast, no direct effect of residents’ participation on satisfaction was detected. Therefore, this study shows that the role of residents’ participation is limited to mediating between health literacy and residents’ satisfaction.

**TABLE 6:** Intermediary effects between factors in the model.

Relationship	Point estimate	Product of coefficients		Bootstrap 95% CI			
				Bias-corrected		Percentile	
		SE	Z	Lower	Upper	Lower	Upper
**Indirect effects**							
HL→P→S	**0.256**	0.042	6.095	0.185	0.353	0.180	0.347
RE→P→S	0.050	0.026	1.923	−0.001	0.100	−0.002	0.098
**Total effect**							
TE	**0.306**	0.039	7.846	0.234	0.388	0.230	0.383
**Contrasts**							
HL-RE	**0.206**	0.058	3.552	0.112	0.343	0.108	0.335

*Statistically significant values are in boldface.HL, health literacy; RE, residents’ expectation; P, participation; S, satisfaction; TE, total effect.*

Residents’ expectation had no direct effect on residents’ satisfaction through residents’ participation. In addition, expectations had no indirect effect either. Thus, our data did not reveal any intermediary influences between residents’ expectations and their satisfaction.

#### Refinement of the Model

**FIGURE 2:**
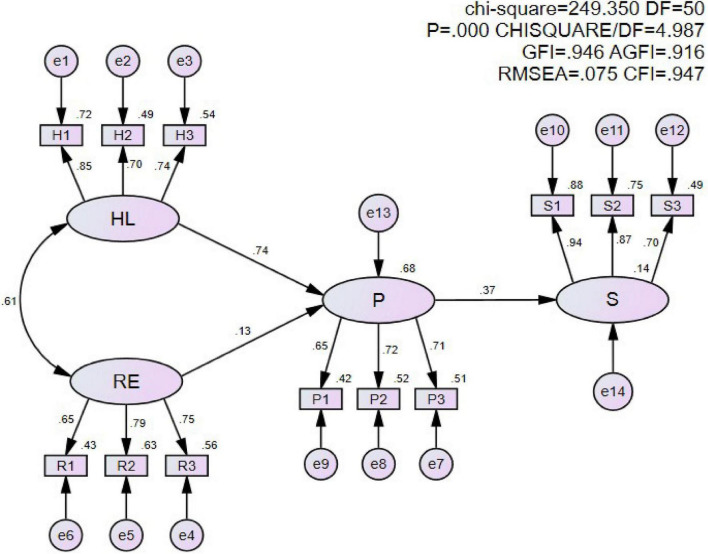
Final structural equation model. In this model, “HL,” “RE,” “P,” and “S” oval boxes are dimension variables; “H,” “R,” “P,” and “S” rectangle boxes are observed variables; “e” boxes are error; The number next to the arrows are parameters in standardized estimates. HL, health literacy; RE, residents’ expectation; P, participation; S, satisfaction.

Based on the hypothesis testing in Table 5, the model was modified to remove the paths associated with H4 and H5 (Figure 2). This final model indicates that urban residents’ health literacy strongly influences their participation in public sports services.

## Conclusion

In this study, a structural equation model was established to determine the main influencing factors affecting urban residents’ satisfaction with public sports services in the “new normal” of daily measures to prevent epidemic resurgence. We showed that health literacy and residents’ expectations are the biggest influencers on satisfaction. In addition, we found that residents’ health literacy greatly influences participation in public sports, and our results reveal the important intermediary role of participation between residents’ health literacy and their satisfaction. These findings are consistent with previous work (Gibney and Doyle, 2017; Shiratsuchi et al., 2021), but the mediating effects described here are novel and lead to concrete recommendations for improving public sports services.

Residents’ changes in mind about health life need to be cared during the COVID-19 Epidemic. However, there is no public sports services model for this situation. The current models paid more attention to objective levels, such as service quality or services types. Therefore, with devising a new model, our study focuses on residents’ health literacy and expectations to meet residents’ need influenced by the COVID-19.

The intermediary role of participation needs to be brought to the attention. Higher participation of urban residents in public sports services directly leads to higher satisfaction, while participation is influenced by and expectations. On the one hand, residents’ personal health literacy has an indirect effect on satisfaction with residents’ participation serving as an intermediary factor. In the proposed model, health literacy can be sub-divided into: having high awareness of physical exercise, having basic health knowledge and concept literacy, and having basic sports skills. Urban residents with strong awareness of all three skills are more enthusiastic about participating in public sports service. This suggests that improving urban residents’ health literacy and sports awareness is an important way to promote their sports participation. And our results indicates that health literacy itself can greatly influence residents’ expectations.

On the other hand, expectations did have an impact on participation. Residents with certain sports expectations will have corresponding needs that they can meet only by participating in sports activities. Further examination of the research results indicates that expectation of providing convenient and high-quality sport facilities should be a significant factor in predicting increasing participation. In fact, previous studies (Ding et al., 2014; Cheng et al., 2016; Zhou et al., 2020) also showed that closer, community-based facilities are often preferable to sport-specific facilities in terms of sport participation. Whether other intermediary variables also affect satisfaction needs further study.

## Recommendations

### Promote the Participation of Urban Residents in Public Sports Services by Improving Their Health Literacy

We defined health literacy as the residents’ awareness of physical exercise, health knowledge and ideas, and sports skills. The COVID-19 had a great influence on public’s health awareness to some degree. The complementary nature of these attributes can be improved through long-term continuous study and practice, thus promoting the participation of urban residents in public sports services.

First, community and government agencies should provide a comfortable physical exercise place for urban residents, and promote the benefits of participation. Second, the community should regularly interview residents about their sports hobbies, physical conditions and leisure time. This information may then be processed by public service providers to help them promote sports preferred by most residents or to adjust service opening times to be more convenient for residents. Third, the community should widely publicize all kinds of health knowledge and ideas through various information platforms so that urban residents can understand the true meaning of health and how to maintain it through physical exercise. Fourth, the community should expand the ranks of “social sports instructors” who takes the responsibility of direct organizers, managers and mentors of community sports and deepen their training, provide related advice services for urban residents, and thereby improve their sports skills and enthusiasm for participating in physical exercise.

### Build and Improve the Platform for Urban Residents to Participate in Public Sports Services

Satisfaction is the subjective evaluation of the gap between the expectation before participating in the public sports service platform provided by the community and the real experience after participating. Therefore, in order to meet residents’ growing demand of sports services and improve residents’ satisfaction during the COVID-19 epidemic, it is necessary to build and improve the corresponding public sports service platform.

Community and government departments should increase investment in public sports services and improve the corresponding facilities and equipment. Second, the public sports service management department should organize and coordinate internal supervision and management mechanisms to ensure that public sports services run smoothly on a daily basis. Third, the community should publicize relevant public sports service information in a timely manner, so that residents can plan ahead. Fourth, the community should provide corresponding public sports guidance and consultation services, so that urban residents can use public sports services properly and safely rather than get sports injury which caused by lacking of warming up or correct use of sports facility. Fifth, the community should provide routine health examination ns to residents to make them aware of any health conditions or to ensure that their physical activities are appropriate for their health condition. Sixth, the community should hold regularly scheduled sports events that allow urban residents to consistently participate.

### Acknowledge Different Expectations of Urban Residents About Public Sports Services

The COVID-19 increased residents’ demand of public sports services with their expectations enhanced. Urban residents’ expectation of public sports service will most likely be influenced by different factors, such as education level, different populations, or gender, resulting in different demands for public sports services. For example, urban residents with a high education level have relatively high expectations for hardware services (venues and equipment) and software services (publicity, consultation, guidance, and sports events). Young and middle-aged people typically participate in public sports services to relieve work pressure and strengthen their physique. Therefore, the public sports service department should provide a comfortable venue and equipment service for high- and low-intensity exercise. In contrast, elderly residents participate in public sports services more for leisure time, self-cultivation and longevity. The public sports service departments may address the needs of this group by providing a variety of low-intensity sports, such as public square dancing, Tai Chi, and qigong for health. Providing sporting opportunities favored by urban residents will greatly improve their participation and satisfaction. At the same time, all types of people can benefit from accessibility to sports health information covering a broad scope of topics and recruitment of highly trained personnel who undergo continuous training to stay apprised of the most up-to-date information and training practices.

## Limitations

The major limitation of the present study is that the potential drawback of the biased sample. The survey was conducted during the COVID-19 epidemic through the form of online questionnaires, resulting in that most of the respondents were young people. Future studies should investigate samples of different age groups of urban residents. The present study was heavily biased toward more young educated participants, so the results may not be generalized to all urban residents. Still, key findings were discovered that can be translated into actionable procedures to improve satisfaction with public sports services.

## Data Availability Statement

The raw data supporting the conclusions of this article will be made available by the authors, without undue reservation.

## Ethics Statement

Ethical review and approval was not required for the study on human participants in accordance with the local legislation and institutional requirements. Written informed consent from the patients/participants or patients/participants legal guardian/next of kin was not required to participate in this study in accordance with the national legislation and the institutional requirements.

## Author Contributions

ML, DY, BC, and ZW have contributed equally to the design and implementation of the research, analysis of the work, and share first authorship. ZC, YP, SG, and ZJ have contributed equally to carried out the experiments, to verify the numerical results, and share second authorship. SL and YJ conceived the study and were in charge of overall direction and planning. All authors discussed the results and contributed to the final manuscript.

## Conflict of Interest

The authors declare that the research was conducted in the absence of any commercial or financial relationships that could be construed as a potential conflict of interest.

## Publisher’s Note

All claims expressed in this article are solely those of the authors and do not necessarily represent those of their affiliated organizations, or those of the publisher, the editors and the reviewers. Any product that may be evaluated in this article, or claim that may be made by its manufacturer, is not guaranteed or endorsed by the publisher.
